# Insights into the Sustainable Development of the Bioeconomy at the European Level, in the Context of the Desired Clean Environment

**DOI:** 10.3390/ijerph191811286

**Published:** 2022-09-08

**Authors:** Delia-Elena Diaconașu, Ionel Bostan, Cristina Căutișanu, Irina Chiriac

**Affiliations:** 1 Department of Social Sciences and Humanities, The Institute of Interdisciplinary Research, Alexandru Ioan Cuza University of Iaşi, 11 Carol I Boulevard, 700506 Iasi, Romania; 2 Centre for European Studies, Faculty of Law, Alexandru Ioan Cuza University of Iași, 11 Carol I Boulevard, 700506 Iasi, Romania; 3 Faculty of Law and Administrative Sciences, Ștefan cel Mare University, Universitatii 13, 720229 Suceava, Romania; 4 CERNESIM Environmental Research Center, The Institute of Interdisciplinary Research, Alexandru Ioan Cuza University of Iaşi, 11 Carol I Boulevard, 700506 Iasi, Romania

**Keywords:** economic-social-environmental trinity, bioeconomy value added, sustainable development, human capital, role of women, informal institutions, panel corrected standard errors estimator

## Abstract

The increasing awareness of the impact of global climate change has brought bio-based projects back into consideration. Thus, having as supports the reality of the troubling scenario that threatens the entire ecosystem and the up-to-date theoretical discourse and debate on sustainable development, this article aims to investigate the socio-economic and institutional determinants that trigger the dynamics of the bioeconomy value added indicator—a valuable instrument developed and recently launched by the EU’s BioMonitor project. Using a panel corrected standard errors framework, we find that investment in human development along with innovation, the growing role of women and sound public governance have a positive effect on the transition towards a durable and resilient bioeconomy at the European level. This naturally implies that a combination of social and technological innovation can ensure the rise of a sustainable bioeconomy.

## 1. Introduction

The Brundtland Report brought to the attention of the entire world the concept of sustainable development, where the key element of its message is the word “ability”. Since then, sustainability has become a buzzword. Among the concepts that have developed alongside the notion of sustainable development, the bioeconomy has gained weight in the minds of academics and practitioners. In this regard, the groundwork for this was laid by Georgescu-Roegen [1,2] who, more than a decade earlier, had prophesied that the global entropy was a zero-sum game and reformulated the economic process as bioeconomics. Yet even though these concepts have infused the quotidian facts, just like in the case of sustainable development, there is no universally accepted definition of the bioeconomy, or consequently no unified measure of it.

On this basis, the existing debates in the literature about the nexus between bioeconomic policy and sustainable development goals, as specified in the UN conventions—a sustainable governance framework for the bioeconomy—do not provide a univocal answer (see, e.g., [3,4,5]). As expected, theoretical arguments on whether bioeconomy leans more towards the weak or the strong form of sustainability have arisen (see, e.g., [6,7,8]). However, regardless of whether one considers the weak or strong type of sustainability, the development of the bioeconomy cannot happen overnight; this is a long-term process that implies a high degree of structural change and adaption and, moreover, should be an inherent part of the economic–social–environmental trinity. Thus, the question that arises is what could harmonize the bioeconomy with the three pillars of sustainability in the near future? One answer at hand—in accordance with the weak sustainability theory—is intellectual capital. According to Ricardo’s logic, machines do not “grow” unless this is together with people, not independently, and, furthermore, there exists a non-conflictual sequential order in which an old machine is replaced by a new one. This Schumpeterian-like creative destruction is neither a snapshot nor occurs for everybody at the same time. Marx’s “general intellect”, science and technical progress infuse not only economics but also society and life in general [9]. Therefore, the man-made capital and the chance to reach progress based on continuity can be seen as solutions at least to relative scarcity and a way of achieving the coveted self-development.

More urgently, at present, the warning of the latest Intergovernmental Panel on Climate Change Reports of 2021 and 2022 [10,11] that it is “very” or “extremely” likely that human influence is the main driver of increased well-mixed greenhouse gas, increased global surface temperature, decreased Arctic Sea ice and increased global mean sea levels and that the world is already certain to face further climate disruptions for centuries to come unless urgent actions to deeply reduce greenhouse gas emissions occur represents a fatalist yet harsh reality. Here, the bioeconomy enters the scene, adopting a leading role in this dramatic perspective. As two of the most important advocates of the bioeconomy at the EU level, Alfredo Aguilar and Christian Patermann, exemplarily state in their attempt to settle a global biodiplomacy, “mankind has two unlimited resources: the energy coming from sunlight and human innovation. Our survival as a species depends on developing the second to guarantee the global sustainability of our planet” [12] (p. 25). This Solow tint perspective seems to us a reasonable equation that encompasses intra- and intergenerational relationships. Overall, hope lies in knowledge, in the creative force of human beings and, as suggested by Vivien et al. [13], in always bearing in mind a green growth model. 

Broadly speaking, in this paper, we aimed to strengthen and reiterate the conviction that the symbiosis of human capital and human-made capital represents the hard core of finding a positive direction to achieve robust, resilient and sustainable development, in which, we believe, the bioeconomy has its well-deserved place. In this respect, we consider that the major prerequisites for creating an effective bioeconomic strategy and, finally, for achieving the metamorphosis of the conventional linear economy into a circular bioeconomy are mainly human development and innovation and technology, along with institutional quality (see also [14,15]). Specifically, the empirical literature emphasizes the importance of human development along with its resultant, human-made capital, in the pathway to a sustainable bioeconomy (see, e.g., [16,17]). Since human activity has led to environmental degradation, it is also up to human ingenuity to fix these urgent problems. In addition, effective, dynamic and good governance also plays a decisive role in surpassing the stringent challenges facing humanity today (see, e.g., [18,19]). Moreover, given the fact that the bioeconomy-related literature abounds in technological, chemical and biological studies but is scarce in terms of the social science perspective (see Sanz-Hernández et al. [20] for a comprehensive review from a social science standpoint), our analysis seeks to contribute precisely to the fact-finding regarding the importance of socio-economic components in sustaining the transition to a bio-based economy. Briefly, the purpose of our paper is to analyze several socio-economic and institutional factors—intellectual capital and social norms of behavior—that drive the dynamics of the bioeconomy value added within the European Union. In this regard, the factors considered in our research are related to investment in knowledge and human capital, the growing role of women and the power of good rules. In other words, we want to study the echoes that human creation and (inter)action have in the development of bioeconomy. 

Here, several remarks require further emphasis. This paper contributes to the related literature in three ways. Firstly, to the best of our knowledge, this is the first study that addresses and analyzes the determinants of the EU bioeconomy value added indicator developed by the BioMonitor project—a valuable instrument that represents a holistic measurement to capture the contribution of bioeconomy to sustainable development in a complete manner. Secondly, by explicitly including in our analysis the role of women and gender equality, we aimed to highlight the fundamental contribution of these drivers to the consolidation of the bioeconomy and its further development. To date, a very limited number of studies have revealed the nexus between the role of women and gender equality and the bioeconomy progress (see, e.g., [21,22]). However, none of these studies emphasized the importance of women and gender equality in creating added value in the bioeconomy sphere. Furthermore, the need for this analysis clearly emerges from the systematic review of the literature on the connection between gender and bioeconomy developed by Sanz-Hernández et al. [23]. Briefly, the authors concluded in a trenchant manner that “bioeconomy literature is making hardly any contribution to the debates and social currents that link gender, development, and sustainability” where “women are simply named”. Thirdly, by encompassing the quality of informal institutions in our analysis, we seek to emphasize how these unofficial norms are connected to the evolution of the bioeconomy (see also [24]). We believe that aggregating a world with the attributes of sustainability cannot be conceived outside the existence of good practices. 

In summary, a picture that captures the socio-economic and institutional causalities of the progress of bioeconomy could be of great importance as there seems to exist a gap between the desired contribution of bioeconomy to the sustainable global development process and the perception of societal stakeholders (see Dieken et al. [25] for an exhaustive literature review). Furthermore, without a better or proper understanding and the involvement of individuals regarding the impact of the bioeconomy-related phenomenon, the risk of jeopardizing sustainable development goals can occur. Still, we do not intend to develop an ideal recipe for the sustainability of bioeconomy because we are convinced that there are no universal models of sustainable development. We only seek to determine some key aspects of the sustainable development of the bioeconomy by tracking down social, economic and institutional features that could facilitate the path to a durable development. Thus, the present study aims to fill these gaps in the literature in order to serve as a guide for all the potential beneficiaries, from the individual consumer to the policymaker, directly or indirectly involved in the triptych of sustainable development that calls for economic growth, social harmony and environmental conservation. 

The remainder of the paper is presented as follows. Section 2 synthesizes the main literature on the topic to provide support for the sketching of the conceptual background. Section 3 presents the data and the empirical model. Section 4 provides the results and corresponding discussions. Section 5 reports the primary conclusions.

## 2. Literature Review

As we stated earlier, even though bio-based society has a long history, the manner in which it is designed today—as a technology-related phenomenon—is rather young (see Salvador et al. [26] for a comprehensive review). This avatar of bioeconomy emerged precisely based on the biotechnology revolution [27]. Thus, compelling evidence (see, e.g., [28,29]) argues that human capital is indissolubly linked to the dynamics of this metamorphosis. Moreover, a successful bioeconomic recipe should also incorporate, alongside the requisite knowledge and skills, a well-structured governance plan that should be properly implemented and access to finance [30,31]. Thus, how a bioeconomic policy should be designed in order to be anchored to the needs of the present and future generations and, eventually, to progress towards the global net sustainability of D’Amato and Korhonen [8] has become a state-of-the-art in the academic field.

In our search for additional rigor for our topic, we subject several papers from the vast existing literature to a synthesis exercise. We knowingly resort to this truncation to argue our hypothesis that the progress of sustainable bioeconomy is strongly linked to human ingenuity, gender neutrality and good rules, as we are convinced that there is still enough room to explore in this emerging field, and to show that there are unexplored or very little exploited areas in the existing works that hold great potential in the effort to achieve the desired sustainable bioeconomy and its related concepts (biotechnology, bio-based economy, bio-ecology, circular bioeconomy, etc.). The various aspects we tackled serve the same purpose: to show that only a combination of social and technological innovation attached to economic development can encourage and accelerate the sustainable development of the bioeconomy.

The Bioeconomy Manifesto of the European Mezzogiorno of Koukios et al. [32] presents 10 theses that aim to develop a new model targeting a sustainable bioeconomy, suitable not only for the five Southern European Union states that collaborated but also for most countries in the world. Briefly, the first six critical steps of this statement represent research and innovation actions as key drivers of bioeconomic development. The last steps proposed by the co-signers point towards acquiring new professional skills and developing a multi-policy framework and an international cooperation plan on bioeconomy strategies. In the same vein, Aguilar et al. [33] suggest that the common features that pave the way towards the progress of bioeconomy are, in order of priority, investments into education and research; encouraging an integrated, dynamic, and innovative industrial environment; support from societies and the interconnection of actors. Of course, in shaping the landscape of the bioeconomy, the behavior of the key character—the entrepreneur—is of great importance [34]. In this regard, Krauss et al. [35] concluded that for the start-up companies in biotechnology, intellectual property represents a “currency of innovation” that enables them to gain credibility and secure investments. 

As can be seen so far, theory and empirical evidence provides a common picture regarding the significance of intellectual capital in the development of the bioeconomy. Logically, the much-needed technologies for the dynamics of this process can only be the result of using human capital. Further, this implies a kind of intergenerational externality where causalities transcend a predetermined horizon of time and where each generation takes over and bequeaths improved and refined skills and competences. Thus, education, knowledge and technological innovations are viewed in unison as primary drivers of long-term, sustained development.

However, what is less articulated by the empirical literature dealing with the transition towards a bio-based economy is the role of women and gender equality. The recent study of Shinbrot et al. [36] represented a strong impulse for our work. Briefly, the authors found that the major challenges that women leaders in sustainable development face are patriarchal hierarchies, gender bias and work–family balance, as well as internal barriers such as self-confidence and differences among women (see also [37,38]). However, as the authors posit, one solution to this issue may come from men, who can serve as important allies to women leaders in this sphere. Hence, solid national and international institutions are needed as key actors to combat discrimination by investing in formal diversity, equity policies and common agendas. In this same context, Hakovirta et al. [39] stated that gender diversity on the boards of directors of bioeconomy companies should be improved, arguing that gender equality contributes positively to company performance and the development of organizational capabilities. In addition, Martínez et al. [40] found that an increased female presence on boards enhances corporate social performance and favors sustainable behavior from companies (see also [41] for a review on women entrepreneurs). At a sectoral level, the analysis of Baublyte et al. [42] pointed out that the forestry business model needs reconsideration and revitalization, as an increased participation of women and a more diverse company culture could provide greater relevance for this sector. These recommendations are in line with those of Kesavan and Swaminathan [43], who highlighted that a pro-women orientation of technology and gender-divide reduction are among the key elements that lay the foundation for sustainable rural development (see also [44]). Overall, the results suggest that the empowerment of women and gender equality play a major role in the promotion and construction of a durable development (see, e.g., [45,46,47]). Given the scarce research in in this particular field, our work seeks to narrow this gap by addressing the symbiotic relationship between the empowerment of women and gender equality, on one side, and the progress of bioeconomy on the other side. By integrating woman’s role in the equation of this process, we wish to raise awareness among all stakeholders about the much-needed structural changes of society—changes that imply embracing a transformed culture where equity and diversity are treated as a natural institution and where discrimination or the degradation of any position can only be known from the pages of history. 

In addition, triggering bioeconomic development in an environment that provides equal chances and wellbeing for all the members of the society, provides proper education and access to resources and fosters innovation cannot materialize without a fair, appropriate institutional framework. The efficiency of formal and informal rules constitutes the driving force of the complex social structure where bioeconomic progress can be attained. Obviously, the entire process implies that these norms coexist and mutually interfere. A negative interaction between the two institutional components results in expansionary transaction costs, thus minimizing potential development and vice-versa. On the one hand, the quality and effectiveness of formal institutions—characterized by flexibility and malleability—make a fundamental contribution to the consolidation of the bioeconomy and its future development. Trenchantly, the authors Goven and Pavone [48] affirm that the development of a cohesive bioeconomy should be perceived as a political project—a promissory construct meant to bring about a particular set of political–institutional changes, arguing this through the need for the mobilization and legitimation of government commitments to establish or strengthen political–economic institutions and policy approaches. As with a number of other studies (see, e.g., [49,50,51]), a well-developed, coherent and agreeable regulatory framework represents a linchpin in the aim to build and foster a flourishing bioeconomy. On the other hand, the informal institutions—featured by a strong inertial character that cannot be deliberately manipulated by human intentionality—represent a gravity center of this transformation process in which the bioeconomy has the potential to become a successful story. In this respect, Rafaty [52] revealed that greater perceptions of corruption are highly and robustly associated with weaker climate policies. In the same vein, Cadoret and Padovano [53] and Mavragani et al. [54] found that high levels of effective governance play a pivotal role in promoting and enhancing environmental performance (see also [55,56]). In this regard, it seems that the level of corruption is the common denominator of environmental studies, therefore making it one of the main examples of an institutional obstacle to the evolution of sustainable development. However, it seems that the question of how governance quality influences the overall bioeconomic sphere remains understudied, thus deserving more attention (see, e.g., [57,58]). Against this background, by incorporating the neo-institutional approach, our work seeks to contribute to the fact-finding related to the impact of the quality of informal institutions on the dynamics of bioeconomy added value. 

In conclusion, it is no wonder that the potential unsuccessful story of the bioeconomy sketched by the National Academies of Sciences, Engineering and Medicine [59] points towards insufficient funding for research and development, an inadequate workforce and ineffective regulatory environment. Therefore, overcoming these barriers represents a way of establishing Industry 5.0, where biologization is the guiding principle of the bioeconomy and where biodigital convergence begins to take shape [60,61,62]. 

## 3. Materials and Methods

### 3.1. The Model

In order to investigate the effects of socio-economic and institutional factors on bioeconomy value added, we estimate a linear cross-sectional time-series model where the parameters are estimated by OLS regression.

Specifically, our baseline model is constructed as follows:

(1) $$\mathrm{logBE}-{\mathrm{VA}}_{\mathrm{it}}=\alpha_{0}+\sum_{k=1}^{2}\beta_{k}X_{k,\mathrm{it}}+\sum_{j=1}^{3}\gamma_{j}W_{j,\mathrm{it}}+\delta_{1}{\mathrm{CC}}_{\mathrm{it}}+\mu_{\mathrm{it}}$$

where i, t denotes country i in year t; logBE−VA is the bioeconomy value added log-transformed; $X_{k}$ is a matrix containing the values for the two considered regressors related to intellectual capital that includes gross domestic expenditure on research and development and the Human Development Index; $W_{j}$ is a matrix containing the values for the three considered predictors related to women’s role (e.g., share of women researchers, percentage of employed women being in managerial positions, Gender Inequality Index); CC denotes the Control of Corruption Index; α, β, γ, δ are the estimated coefficients of interest and μ is the error term. 

### 3.2. The Dataset

We focus our analysis on the dynamics of the bioeconomy value added indicator in 19 EU countries. The time frame for our study covers the period between 2005 and 2015. The time span of our panel ends in 2015 because the DataM platform of the European Commission’s Joint Research Center (https://datam.jrc.ec.europa.eu/datam/mashup/BM_BIOECONOMIC_SHARES/, accessed on 1 February 2022), our data source for the dependent variable, provides data only up to this point in time. The reason for taking Austria, Germany, Luxembourg, Sweden and Greece out of the sample is related to the large amount of missing data for one of the independent variables, i.e., the share of women researchers. In addition, due to both the short sample span and the very high values of the dependent variable registered in Spain, France and Italy compared to those recorded by the other EU states, we excluded these three countries from our analysis in order to maintain a reasonable degree of sample homogeneity for our panel analysis. Therefore, the 19 EU countries under investigation are Belgium, Denmark, Finland, Ireland, Netherlands, Bulgaria, Cyprus, Czech Republic, Estonia, Croatia, Hungary, Lithuania, Latvia, Malta, Poland, Portugal, Romania, Slovak Republic and Slovenia. 

### 3.3. The Variables

The reason we chose the total value added of the bioeconomy (BE-VA) developed by Cingiz et al. [63] under the auspices of the BioMonitor project as our dependent variable is twofold. Firstly, this indicator provides valuable insights about measuring the contribution of bioeconomy towards sustainable development using the well-known Input–Output approach, which describes inter-sectorial relationships within an economy (see also [64,65]). Then, the attempt of Cingiz et al. [63] to assess the added value of the bioeconomy in a comprehensive manner by incorporating both fully bioeconomy-based and partly bioeconomy-based industries makes this indicator robust and an important frame of reference for further empirical investigations in a uniform manner at the community level.

To better capture the influence of socio-economic and institutional features on the bioeconomy, we group our explanatory variables into three dimensions that quantify the performance of intellectual capital (X), women (W) and quality of governance. The set of variables that reflects intellectual capital X encompasses the gross domestic expenditure on research and development (GERD) and Human Development Index (HDI). In keeping with the vast body of literature (see, e.g., [28,30,66]), we posit that investment in research represents an appropriate and comprehensive manner to measure creative work and scientific knowledge engaged in promoting the sustainable development of the bioeconomy. As a proxy metric for quantifying human resource and human wellbeing, we used the Human Development Index—a measure often used to assess socio-economic development and analyze its relationship with environmental sustainability [67,68]. The HDI captures the key dimensions of human development, i.e., life expectancy, school enrolment, literacy and the standard of living. As a parenthesis, we point out that the reason for not considering the frequently used GDP per capita variable in our analysis is twofold. Firstly, this traditional indicator only reflects economic development, and we aimed to articulate the impact that both economic and social wellbeing have on the progress of the bioeconomy. Secondly, a growing number of studies found a vexatious negative or neutral causal relationship between economic growth and renewable energy deployment at the European level (see, e.g., [50,53,69]). 

The representative indicators of vector W, which comprises women’s role in the development of bioeconomy, are share of women researchers (W_res), percentage of employed women being in managerial positions (W_mng) and Gender Inequality Index (GII). We argue that the proportion of women as suppliers of knowledge and that have a high-ranking position, along with a balanced gender participation, captures relevant information related to women’s progress and its contribution to the rise of the bioeconomy. 

Finally, the quality of governance is proxied by the Control of Corruption (CC) index. The dynamics of representative institutional arrangements could easily be blamed or praised for failure or success of the bioeconomy project. This social disease, corruption, can erode the transition to a bio-based economy by introducing uncertainty and disorder, which work entirely against sustainability. 

To allow the interpretation of the estimated coefficients as elasticities, we log-transformed the original data, except for the variables expressed in shares and Control of Corruption that had negative values. Several descriptive statistics of the transformed data for the analyzed timespan along with the unit root test are reported in Table 1.

As can be observed from Table 1, several cross-sectional units have a different sample size, and therefore, we perform our analysis in an unbalanced panel framework. In addition, the standard deviation values are quite large, which implies that the cross-country variation of the indicators is not too close to its average value. In line with Hlouskova and Wagner [70], who demonstrated that when dealing with small T and intercepts under stationarity, the best power behavior is displayed by the Levin–Lin–Chu panel unit-root test, we performed this test on all the variables considered in the study. As can be easily seen in Table 1 and as expected—given the fact that the data covers a limited period of time—the null hypothesis that all the panels contain unit roots is rejected at the 1% level—a result that can be interpreted, according to the qualified and balanced suggestion of Pesaran [71], as evidence that a statistically significant proportion of the units are stationary.

### 3.4. Methodology

For the empirical analysis, the legitimacy of the estimations was evaluated by conducting a battery of tests. Firstly, we began our analysis by estimating Equation (1) within a country fixed effects framework, given the longitudinal nature of the database. Then, we applied the Hausman test to decide between fixed vs random effects and the results of the test validated the latter case as the suitable estimator, for the time being. Thirdly, the modified Wald test for group-wise heteroskedasticity supported the existence of heteroskedasticity—a fact that can significantly influence standard errors. This result indicates that the analyzed countries have evolved in different ways, although they are members of the EU community. Fourthly, the cross-section dependence test of Pesaran [72] indicated considerable evidence of residual dependence. The results of these tests are not provided here but are available from the authors upon request. In this context, although the Hausman test has indicated the REE as a suitable estimation method, the presence of group-wise heteroskedasticity and cross-sectional dependence makes the application of this estimator unsuitable. Moreover, the small amount of panel data (T < N) and unbalanced framework are additional challenges present in the data of this study. Therefore, Beck and Katz [73] suggested that the PCSE approach is suitable for mitigating and controlling all these issues, producing relatively efficient parameter estimates (see also [74,75,76] for the use of the PCSE approach in environmental analysis). Thus, in accordance with these studies, we estimated Equation (1) by ordinary least squares using the PCSE technique. As a parenthesis, the PCSE estimator allows the error term μ from Equation (1) to be heteroskedastic, to be correlated over the countries and to follow a first-order autoregressive process over time [77,78]. 

## 4. Results and Discussion

The results of the PCSE models employed to reveal the influence of the socio-economic and institutional factors described in Section 3.3 on the dynamics of the value added of the bioeconomy are reported in Table 2. As the figures in Table 2 indicate, the null hypothesis of the Wald Chi-square test is rejected in all estimated models, indicating that all coefficients of the explanatory variables taken jointly are significant. In addition, the results of all the employed models are statistically significant, except for the human development index in the third model. Briefly, apart from the Gender Inequality Index, all the variables are positively related to the dependent variable.

### 4.1. The Role of Human Capital

The results presented in Table 2 suggest that the knowledge and wellbeing of human capital contributed significantly to the development of the bioeconomy in the examined interval. First, these results are validated by the strategy adopted at the level of the European Union. In this sense, the revised and refined form of the EU Bioeconomy Strategy specifies, under the headings of the three-tiered action plan, the key elements—improving access to finance, deploying targeted financial instruments, e.g., the Circular Bioeconomy Thematic Investment Platform, and promoting education, training and skills across the bioeconomy—that can increase the sustainability and circularity of the bioeconomy. 

Firstly, as expected and unanimously demonstrated by previous literature (see, e.g., [32,66,79]), investment in research and development constitutes a crucial driver of bio-based innovation. Obviously, research and development expenditure and bioeconomy value added follow quite similar paths over the analyzed timeframe. More precisely, both trends describe a sinusoidal shape that shows large increases before a crisis—the financial and European debt crisis—sharp decline during turmoil and moderate recoveries afterwards. What is of interest in this dynamic is that the Baltic states Romania and Slovakia registered the most notable increases of both indicators during the analyzed period, while Finland, Denmark and the Netherlands recorded some mild, modest increases in this respect (bearing in mind that, here, we refer to the relative values of the indicators). A possible explanation for this sharp increase in the BE-VA in the CEE countries could be related to the fact that, in this area, the land biocapacity is still less exploited [80]. Even so, in this region, it seems that most of the labor force works in low-productivity sectors of the bioeconomy compared to the western and northern parts of the continent [65]. In the context, if we consider the absolute value of bioeconomy value-added, we can clearly see that the highest values of the indicator in the analyzed countries are recorded in the Netherlands, Poland, Belgium and Ireland. This picture is somewhat similar to that of D’Adamo et al. [81], who found that the most “virtuous” group in terms of the socio-economic performance of the bioeconomy includes Ireland, Denmark and the Netherlands. Furthermore, an analysis of the R&D expenditure by source of funds and R&D intensity shows a similarpicture. In this regard, within the most research-intensive countries, such as Finland, Denmark, Belgium and the Netherlands, the business sector represents the most prominent R&D performing sector, while in the least research-intensive countries, such as the Baltic states, Slovakia and Romania, the public sector is the main provider of financial resources. Although both public funding and private investment are of great importance in shaping a sustainable bioeconomy, it is only the latter that represents the main force of persuasion and perseverance towards a sustainable and resilient Europe. In the same vein, Ronzon et al. [65] found high heterogeneity in the national bioeconomies across the EU in terms of labor productivity. Obviously, the solution proposed by the authors to reduce the east–west productivity gap mainly concerns the consolidation of research and innovation in the CEE countries. Thus, Europe, and in particular its eastern part, should mostly increase the private investment in research and development in relevant research areas to enhance its competitiveness, import new innovations, productivity, resource efficiency and absorption of innovations, all while shifting towards a durable, climate neutral economy—a reality also confirmed by the BIOEAST initiatives, which involve strengthening research and innovation in CEE countries. Then, the private investments must be complemented with, but not substituted by, the involvement of public funds.

Secondly, the positive nexus between human development and bioeconomic progress is an accurate representation of our anticipated relationship. In other words, greater levels of education, higher incomes and better health promote bioeconomic process at the European level. There is no doubt about the role of education in raising the awareness of individuals and promoting the transition to a sustainable bioeconomy [33,82]. The transformative knowledge provided in higher education institutions can support and enhance the interactions among multiple actors, from producers to consumers of bio-based output, and, moreover, could represent a long-term commitment to the necessary and much-desired change in society that craves to embrace the concept (in fact, a true paradigm) of the bioeconomy. In addition, we are convinced that prosperity supports the development of bioeconomy but not in a random manner. We argue that income inequality can raise serious problems. In this respect, the Gini index has moderately improved in Europe over the analyzed period, but the coefficient still extends over a relatively wide interval, ranging from 0.25 in Slovenia, Slovakia and the Czech Republic to over 0.38 in Bulgaria, Lithuania and Romania. Furthermore, a sustainable bioeconomy is not just about environmental policies but also about a just society. A country where income inequality and poverty are high limits the access to the consumption of bio-based products due to the still high costs of these products. The solution to this issue lies in education. In a sequential order, consolidating this natural institution and fostering and strengthening a competitive entrepreneurial environment, infused with highly skilled human resources, could mitigate income disparities by creating jobs in high-productivity sectors that could, in turn, accelerate the deployment of the bioeconomy [65]. 

Interestingly, we should note that after adding Control of Corruption as a control variable, the coefficient of HDI becomes statistically insignificant. This finding is not quite in accordance with the orthodox perception (see [83,84]). This ambiguity reflected by our results is to an extent in line with the findings of Bourcet [69], Cadoret and Padovano [53] and Sebri [85], who argued that higher income might lead to higher renewable energy consumption (a proxy for bioeconomy), which may offset the income effect, and that the causal relationship between the bioeconomy and income could depend on the country’s level of development. 

### 4.2. The Role of Women Empowerment

According to the estimates in Table 2, promoting women in research and leadership positions contributes to building a sustainable bioeconomy at the European level. This result is also reinforced by the negative impact that gender inequality has on the harmonious development of the bioeconomy. Similar results attesting to the positive impact of gender empowerment in shaping the sustainable economic realm were also found by Shinbrot et al. [36] and Hakovirta et al. [39]. On average, in the analyzed period, the highest percentages of women as suppliers of knowledge and in professional managerial levels are registered in the former communist countries—possibly a legacy of the gender-neutral policy required by the communist ideology.

This much-needed social innovation is congruent with the growing welfare of individuals and represents a transformation that goes shoulder to shoulder with social restructuration and even social destruction (in a Schumpeterian sense), such as renouncing values and behaviors such as gender discrimination, which now should be condemned to dysfunction or inadequacy. At the EU level, the synergy between the European Green Deal and the Gender Equality Strategy—a strategy adopted a little late—could foster the transition to a climate-neutral economy by boosting women’s empowerment, gender equality and social inclusion.

Broadly speaking, we argue that one of the solutions to the most urgent global social and ecological challenges is a combination of social and technological innovation. However, for social innovation to create value for society, the ongoing commitment of sound national and international institutions should not be discretionary.

### 4.3. The Role of Corruption

Finally, the empirical evidence presented in Table 2 suggests that less corruption positively affects the bioeconomy value added indicator. This conclusion backs up previous research [52,54,86] that revealed a negative connection between the transition to renewable energy and government inefficiency. The analysis of the data indicates that the ex-communist countries are characterized by a high level of corruption—another legacy of the communist regime—and the evolution of this indicator over the studied time interval is quite modest. Sustainable development is intended to be based on good practices and strong and effective institutions. Institutions configure and influence the process of transition towards a bio-based economy. Moreover, the entrepreneurial framework and perceived opportunities to innovate in this green field must benefit from the emulating context of effective and stable institutional arrangements to achieve its goals.

## 5. Conclusions

It is noticeable that European countries are developing and expanding their bioeconomies. However, the present policies have a challenging task—one of orchestrating social peace within and between generations, which, of course, is unthinkable without economic peace (and the other way around) and reconciling this in tandem with the boundaries and finiteness of the planet. In this regard, the ever-growing interest and prioritization manifested through actions taken under the EU Bioeconomy Strategy of 2012 and 2018 are straightforward signs of the significant weight of the bioeconomy in promoting and achieving a sustainable, circular and low-carbon economy at the community level. How these strategies can succeed is a very important yet difficult question since there is no universal model to be implemented in order to attain a sustainable, circular bioeconomy. The creative force of human beings and suitable and coherent formal and informal institutions (which, of course, are made by people) are, in our view, two crucial factors that can fulfil this long-lasting process. In a more parsimonious manner, our empirical analysis confirmed our expectations related to the positive effect of investment in human capital and innovation, gender equality and good practices—in this order—on the development of a sustainable bioeconomy at the European level. These findings are now increasingly important to support Europe’s recovery from the COVID-19 crisis, encouraging green, circular and digital transitions of the bioeconomy in an inclusive manner. 

Our analysis has two main strengths: as far as we know, it is the first study to address the complex and complete bioeconomy value added indicator developed by the BioMonitor project, and it is among the few studies to examine the role of promoting women in the bioeconomy sphere. In addition, our analysis exhibits three main caveats: (1) the small number of states and limited time frame covering the period 2005–2015, (2) the limited number of variables included in our analysis in order to preserve the degrees of freedom and (3) the existence of missing data in the case of gender-related variables. In this context, and especially in view of the recent COVID-19 crisis, future research could consist of extending the period of analysis, incorporating other developed and developing countries or integrating other social–economic factors such as labor productivity, entrepreneurial environment and the controversial GDP into the matrix that defines the durable development of the bioeconomy and circular bioeconomy, respectively.

## Figures and Tables

**Table 1:** Summary statistics, source for the analyzed variables and Levin–Lin–Chu panel unit root test results.

Variable	Source	Obs.	Mean	Std. Dev.	Min	Max	LLC
**logBE-VA**	DataM dashboard	209	3.93	0.53	2.57	4.81	−5.10 ***
**logGERD**	Eurostat	209	3.30	1.03	1.43	5.67	−2.63 ***
**logHDI**	UNDP–Human Development Reports	209	−0.06	0.02	−0.12	−0.03	−4.69 ***
**W_res**	Eurostat	144	38.84	8.04	24.00	57.70	−6.13 ***
**W_mng**	Eurostat	133	32.11	6.56	12.20	48.80	−4.78 ***
**logGII**	UNDP–Human Development Reports	209	−0.83	0.25	−1.35	−0.04	−9.24 ***
**CC**	World Bank–WGI database	209	0.88	0.77	−0.26	2.47	−2.73 ***

Notes: LLC test presents empirical statistics of the Levin–Lin–Chu panel unit root test (AIC criteria). *** Indicates significance at 1% level.

**Table 2:** Results from panel analysis estimated with PCSE.

Dependent Variable: logBE-VA				
Independent Variables		Model (1)	Model (2)	Model (3)
**Vector X (Human capital variables)**	logGERD	0.368 *** [0.002]	0.406 *** [0.015]	0.409 *** [0.016]
	logHDI	3.012 *** [0.208]	4.441 *** [1.699]	1.634 [1.268]
**Vector W (Role of women variables)**	W_res		0.022 *** [0.002]	0.021 *** [0.002]
	W_mng		0.005 * [0.003]	0.006 ** [0.003]
	logGII		−0.392 ** [0.166]	−0.340 ** [0.155]
**Quality of governance variable**	CC			0.094 *** [0.029]
	Cons	2.922 *** [0.014]	1.537 *** [0.206]	1.305 *** [0.179]
	N	209	127	127
	R^2^	0.572	0.637	0.641
	$\mathrm{Wald}\;\left(\chi^{2}\right)$	41,767.06 ***	3533.63 ***	6131.78 ***

Notes: The Wald test verifies the null hypothesis of non-significance of the set of parameters in the. model. Panel corrected standard errors are reported in squared brackets. *** *p* < 0.01, ** *p* < 0.05, * *p* < 0.1.

## Data Availability

Not applicable.
